# The Gene Expression Barcode 3.0: improved data processing and mining tools

**DOI:** 10.1093/nar/gkt1204

**Published:** 2013-11-22

**Authors:** Matthew N. McCall, Harris A. Jaffee, Susan J. Zelisko, Neeraj Sinha, Guido Hooiveld, Rafael A. Irizarry, Michael J. Zilliox

**Affiliations:** ^1^Department of Biostatistics and Computational Biology, University of Rochester Medical Center, Rochester, NY 14642, USA, ^2^Department of Biostatistics, Johns Hopkins University, Baltimore, MD 21205, USA, ^3^Informatics and Systems Development, Loyola University Stritch School of Medicine, Maywood, IL 60153, USA, ^4^Nutrition, Metabolism & Genomics Group, Division of Human Nutrition, Wageningen University, The Netherlands, ^5^Department of Biostatistics and Computational Biology, Dana Farber Cancer Institute, Boston, MA 02215, USA and ^6^Center for Biomedical Informatics, Department of Pharmacology and Molecular Therapeutics, Loyola University Stritch School of Medicine, Maywood, IL 60153, USA

## Abstract

The Gene Expression Barcode project, http://barcode.luhs.org, seeks to determine the genes expressed for every tissue and cell type in humans and mice. Understanding the absolute expression of genes across tissues and cell types has applications in basic cell biology, hypothesis generation for gene function and clinical predictions using gene expression signatures. In its current version, this project uses the abundant publicly available microarray data sets combined with a suite of single-array preprocessing, quality control and analysis methods. In this article, we present the improvements that have been made since the previous version of the Gene Expression Barcode in 2011. These include a variety of new data mining tools and summaries, estimated transcriptomes and curated annotations.

## INTRODUCTION

Until the publication of the Gene Expression Barcode (Barcode), databases using publicly available microarray data were limited to biological questions based on measures of relative gene expression (1,2). They were unable to answer the most fundamental question—which genes are expressed in a given sample. The Barcode was the first database to report reliable estimates of absolute gene expression, allowing an approximation of the human and mouse transcriptomes.

Determining the genes expressed for every tissue and cell type in the body has important consequences for basic cell biology, generating hypotheses for gene function and studying transcriptional changes in disease. Gene expression signatures have been used to make clinical predictions in a number of cancers and to elucidate basic gene expression biology. With the advent of high-throughput technologies, addressing these issues has become more feasible, although several technological and statistical challenges remain. Now in its third generation, Barcode 3.0 has made several improvements to its implementation, greatly expanded its database and improved the web tools to help researchers better investigate the human and mouse transcriptomes.

High-throughput studies are hampered by false-positive results, which can mislead researchers and lead to irreproducible results (1,3). The barcode algorithms were designed to help minimize the impact of false positives (positive results due to technical artifacts and not biology) on gene expression studies, particularly for those focused on finding biomarkers for diseases. We continue to improve the algorithms and results, with the ultimate goal being a complete molecular description of the genes expressed in each cell type in the body. Toward this end, we have downloaded all of the publicly available microarray data from six of the most used platforms and analyzed it using a novel suite of statistical tools designed to obtain meaningful information from a single gene expression experiment, while minimizing the number of false positives (2,4,5).

## DATABASE METRICS

Table 1 shows the data and platform improvements for Barcode 3.0. We have updated the barcodes for the original and most popular Affymetrix platforms, U133A, U133 plus 2.0 and MOE 430 2.0. In addition, we have added data from three new platforms, U133A 2.0, Human Gene 1.0 ST and Mouse Gene 1.0 ST. This extends the barcode technology from the 3′ IVT (*in vitro* transcription) arrays to the whole gene arrays. Owing to limited data and statistical challenges, barcodes for exon level data will be part of future releases.

**Table 1.** Barcode database metrics

Affymetrix GeneChip	GEO^a^ platform ID	Barcode 2.0 sample number	Barcode 3.0 sample number
U133A	GPL96	13 824	23 936
U133 plus 2.0	GPL570	18 656	63 331
U133A 2.0	GPL571	0	8528
Human Gene 1.0 ST	GPL6244	0	10 309
MOE430 2.0	GPL1261	9652	32 241
Mouse Gene 1.0 ST	GPL6246	0	10 505

^a^Gene Expression Omnibus (6).

## IMPROVED SAMPLE CURATION

Although the majority of databases that use publicly available gene expression data rely on the curation supplied to GEO and ArrayExpress by the experimenter, we manually curate a vast amount of the publicly available data. A large problem with the public microarray databases is their use of an open vocabulary and open submission structure. This hinders computational approaches to curation and requires an extensive manual curation effort to acquire the data necessary for the barcode models. Currently, the annotation data are collected and parsed to provide the most useful text fields. Then biological researchers manually identify normal and tumor samples for parameter estimation. The manual curation also determines whether the sample is a tissue or a purified cell type. Currently, only fluorescence-activated cell sorted or laser-capture microdissection isolated samples are considered purified cell types.

## IMPROVED QUALITY CONTROL

Shortly after the publication of Barcode 2.0, a single-array measure of quality was developed and used to show that ∼10% of publicly available HGU133a and HGU133plus2 microarray data are of poor quality (5). In Barcode 3.0, we use this measure of quality to filter poor quality arrays when estimating the barcode parameters, resulting in improved estimates of the null mean and variance. Furthermore, these quality metrics are made available for all arrays via the Barcode 3.0 website, allowing the user to set their own quality threshold.

As high-throughput technologies are prone to outliers and batch effects, it is important to limit these sources of error. The barcode methodology is inherently conservative in its estimation of absolute gene expression (to be called expressed, a gene is required to be five standard deviations above its null mean). Furthermore, discretization has been shown to greatly reduce the influence of batch effects (1).

## UPDATE OF BARCODE 2.0 PLATFORMS

The Barcode 2.0 database contained data from the three most widely used Affymetrix microarray platforms—U133A, U133 plus 2.0 and MOE 430 2.0. Since then, the amount of publicly available data has nearly tripled (Table 1). In addition to the improvements in quality control described earlier in text, the increase in input data led to improved estimates of the barcode parameters, and therefore improvements in the estimation of absolute gene expression.

Compared with Barcode 2.0 parameter estimates, the Barcode 3.0 estimates were fairly similar. Estimates of the null means were highly correlated between version (GPL96: 0.99, GPL570: 0.98, GPL1261: 0.98) and only ∼1% of the null means differed by >1. However, there were a few genes whose null mean changed by >2 between versions. There are two potential reasons that this change could have occurred: (i) the Barcode 2.0 estimate was driven by a handful of poor quality arrays that have been removed from the training data in Barcode 3.0 or (ii) the additional training data used in Barcode 3.0 provided a more accurate estimate of the null mean. This suggests that although Barcode 2.0 performed well, there are significant improvements that can be made by improved quality control and incorporation of additional data.

## NEW BARCODE 3.0 PLATFORMS

In addition to updating the microarray platforms present in Barcode 2.0, we have added three new platforms—U133A 2.0, Human Gene 1.0 ST and Mouse Gene 1.0 ST. The latter two represent a newer generation of Affymetrix microarray that contains probes designed to target the entire gene sequence rather than only the 3′ end. Preprocessing of these arrays requires a slightly different fRMA implementation that includes both probe-effect and exon-effect parameters to distinguish between batch-effect susceptible probes and probes targeting exons involved in alternative splicing (7).

## BOTTOM-UP RESEARCH

Although a global approach to understanding gene expression is critical, it is also important to make this wealth of data available to researchers using bottom-up approaches to studying gene expression—i.e. looking at one or a handful of genes at a time. To facilitate this kind of research, we have designed a new suite of data mining and analysis tools. These tools will allow researchers to query the database for changes at the individual gene level so their research can be focused and not overwhelmed by large numbers of extraneous results. This approach requires some additional considerations, such as the reliability of each probe set, which we have provided in graphical form.

**Figure 1.**
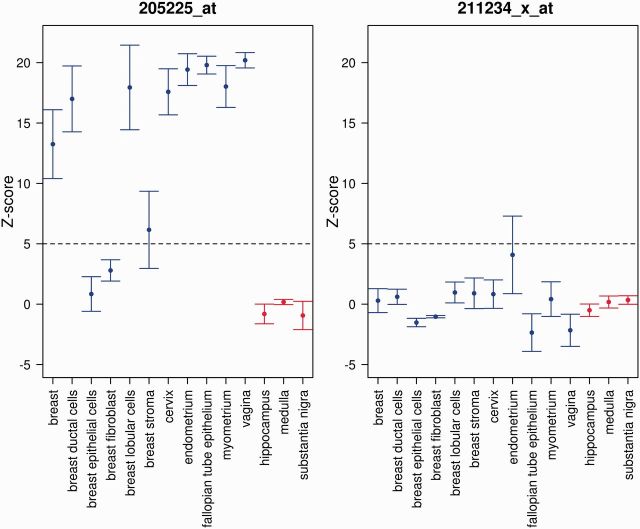
Across-tissue expression for two probe sets for estrogen receptor 1. 205225_at. 211234_x_at. Shown are the *z*-scores ± the median absolute deviation (MAD). A *z*-score >5 suggests the gene is expressed in that tissue. The figure also demonstrates how purified cells give improved results as breast tissue shows ESR1 expression, but the purified cell types show it is restricted to ductal cells, lobular cells and stroma (Figure 1, 205225_at). Blue–female reproductive tissues/cells. Red–brain tissues. Instructions for reproducing the figures are provided in the Supplementary Material.

The first step toward allowing researchers to query the database from a bottom-up approach is to determine how well each probe set on an array works. The probe sets work with varying efficacy and this is an important consideration for the researcher before trying to interpret any results across studies. To aid in this evaluation, we provide the user with the across-tissue distribution of gene expression, a measure of probe set reliability (average entropy), and a probe page to enable sharing among researchers. An example is shown with estrogen receptor 1(ESR1), a common marker used in breast cancer research. There are nine probe sets for ESR1 on the Affymetrix U133 plus 2.0 microarray platform. When examining their across-tissue distribution, few of the probe sets were estimated to be expressed in any given tissue. A *z*-score >5 is considered to be evidence of expression, and only one probe set achieved this expression in a variety of tissues, 205225_at (Figure 1 and Supplemental Material). Among the other eight probe sets, no tissue showed a median *z*-score >5 (Figure 1b). This is strong evidence that only one of these probe sets, 205225_at, can measure ESR1 expression.

**Figure 2.**
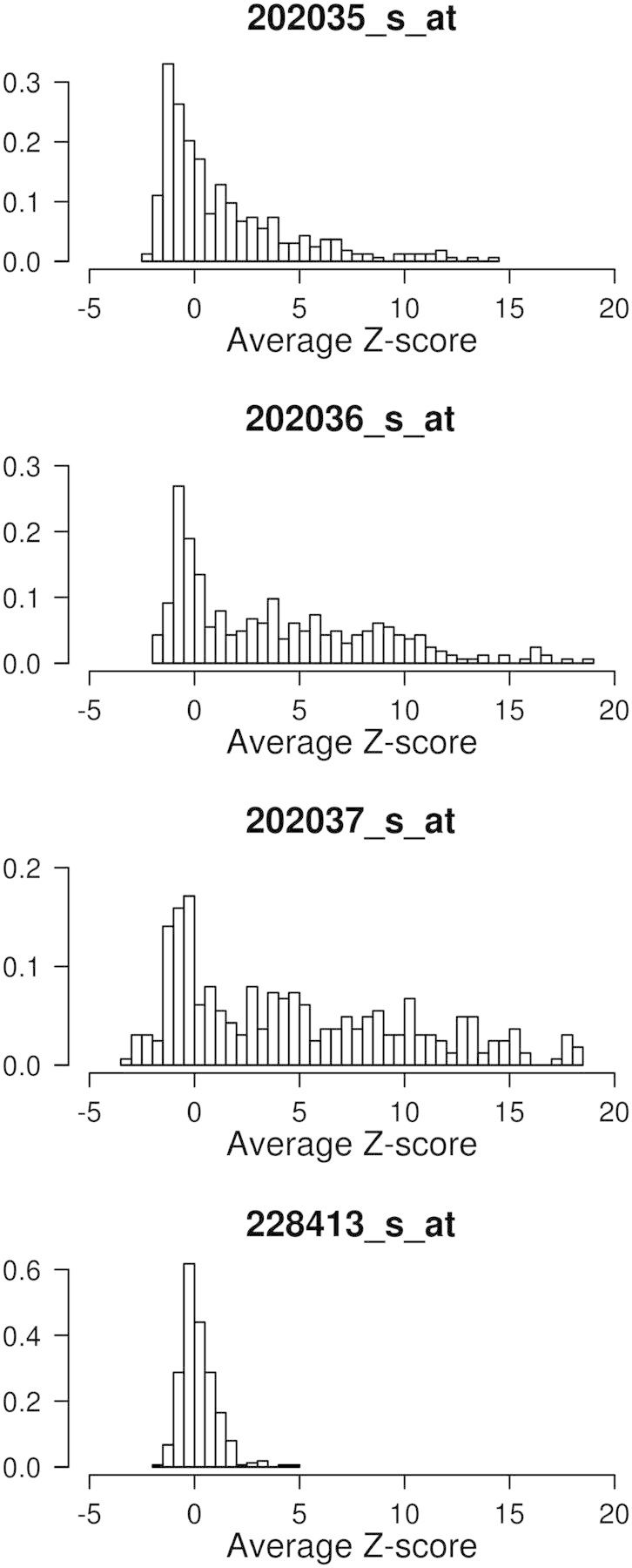
The distribution of average *z*-scores across tissues and cell types for four probe sets mapping to the gene SFRP1. The first three probe sets show expression of SFRP1 in a fair number of tissues/cell types; however, the fourth probe set does not exceed the expression threshold in any tissue or cell type. Figures such as this one can be used to evaluate the performance of multiple probe sets that map to the same gene by comparing the proportion of tissues/cell types in which the gene is called expressed, the dynamic range of the standardized expression estimates (*z*-scores) and the separation between the unexpressed null distribution and the expressed signal.

Another consideration is that even though two probe sets may both successfully measure gene expression, they might not have equal dynamic ranges. A good analogy is antibodies used for flow cytometry experiments. Some antibodies show a strong signal and clearly differentiate a positive binding event from background. Similarly, probe sets show different binding behaviors due to physical characteristics, such as GC content. By examining the distribution of average *z*-scores across tissues and cell types, one can easily compare the ability of different probe sets to detect gene expression. For example, GPL570 has four probe sets that map to the gene SFRP1 – 202035_s_at, 202036_s_at, 202037_s_at and 228413_s_at. Looking at their across tissue and cell type expression distributions, one can clearly see that 228413_s_at does not detect a significant level of expression in any measured tissue or cell type; however, the other three probe sets all detect expression in a sizeable number of tissues (Figure 2). Although each of these three probe sets has a clear null distribution (left-most mode) and a range of gene expression signal (long right tail), probe sets b and c have a larger dynamic range than probe set a. However, none of these probe sets show a clear separation between the background distribution and the expressed distribution.

Two different search methods have also been added. First, a researcher can identify the genes and experiments of interest and directly download the preprocessed data for analysis, using proven statistical methods (8). Alternatively, the website provides consensus data for tissues and purified cell types that can be downloaded and compared, such as normal breast and breast tumors. As with all experiments, the results from *in silico* data mining should be considered preliminary and validated through independent experimentation. It is important for researchers to carefully consider the potential confounding effects from false positives due to batch effects. One method to examine potential false-positive results is to graph the Affymetrix control probe sets (dap, thr, phe and lys) along with the gene of interest. (Currently, the full probe set names must be used on the website, AFFX-DapX-3_at, AFFX-ThrX-3_at, AFFX-PheX-3_at and AFFX-LysX-3_at).

## PATIENT-SPECIFIC (SINGLE-ARRAY) RESULTS

**Figure 3.**
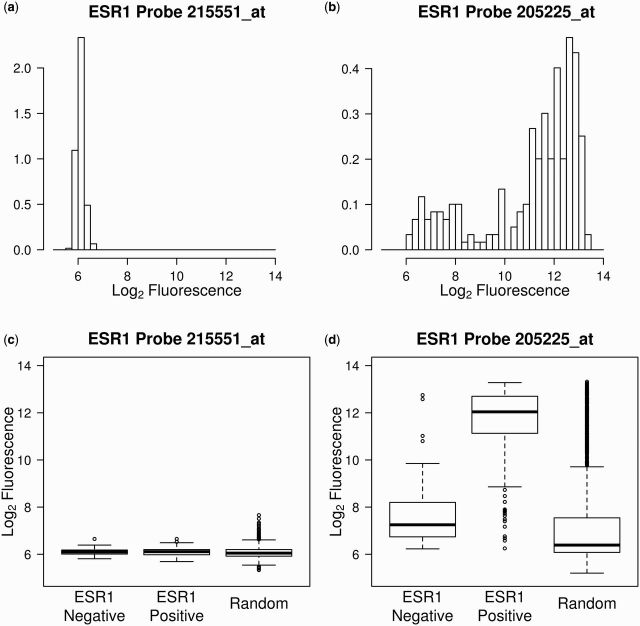
Log_2_ fluorescence values, after frma preprocessing, are shown for patients from GSE3494 (**a**) probe set 215551_at, a probe set which does not accurately measure ESR1 expression and (**b**) 205225_at, a probe set which performs well. (**c**) Log_2_ fluorescence correlated with RT-PCR status and 2000 random normal tissues. Probe sets 215551_at and (**d**) 205225_at, which correspond well to ESR1 status. ESR1 status was determined by RT-PCR in Miller *et al.* (9).

One of the primary benefits of the barcode approach is the ability to obtain patient-specific (single array) results. Because the barcode algorithm draws power by analyzing the across-sample distributions, meaningful results can be obtained from a single array. This is particularly important in clinical research, where each patient may be unique and combining data can dilute important differences. The barcode algorithm is designed to provide this type of data so that patient-level expression data can be meaningfully interpreted. An example is shown in Figure 3, again looking at ESR1. Most studies determine ESR1 status using an alternative approach, such as RT-PCR and then lump ESR1+ and ES1R- patient samples together to find gene expression signatures. However, an alternative approach would be to look at each sample independently, determine ESR1 status and look at other genes of interest for that sample. This approach eliminates the potential bias introduced by precategorizing patients and allows more differences to be determined, as samples are not pooled to gain statistical strength.

Figure 3 shows how this can be accomplished. Figure 3a and b show histograms for the measured fluorescence from two ESR1 probes for the patients from Miller *et al.* (9). As discussed previously, only 1 ESR1 probe detects ESR1 expression, 205225_at, and it shows two clear distributions (Figure 3b). In contrast, probe 215551_at clearly does not work. Figure 3c and d show that the results at the patient level correspond well with RT-PCR results for the probe set that works for ESR1. Therefore, a researcher could separate the microarray results into ESR1− (<8 log_2_ fluorescence) and ESR1+ (>10 log_2_ fluorescence) and extend this result to any other gene or genes of interest using the barcode database to further subdivide this patient population.

## DISCUSSION

With the completed human and mouse genomes, researchers are now thoroughly studying the transcriptome, epigenome and proteome. As a reliable approximation of the transcriptome, the Barcode data have been used to compliment epigenetic studies (10), to improve ChIP-seq and ChIP-chip data analysis (11) and to investigate increased heterogeneity in cancer (12). The barcode data are an integral part of the EpiViz webtool, which links transcriptomic and epigenomic data—an example workspace can be seen at http://epiviz.cbcb.umd.edu/?workspace=0271BFB50384DE1DB4A3D712702D0E34. As the genome, epigenome and proteome all interact with the transcriptome, the barcode estimations will be of interest to a broad community of researchers. The frma R/BioC package, coupled with the frmavecs data packages for each supported platform, allow one to easily incorporate barcode data into one’s own analyses (4,7).

The two primary bottlenecks to supporting additional high-throughput platforms are as follows: (i) access to enough publicly available data and (ii) manual curation of the data annotation. Increased journal requirements to make data publicly available and online repositories such as GEO and ArrayExpress have reduced the first bottleneck considerably. For most widely used platforms, sufficient data are made publicly available within the first 2–3 years of use. However, little has been done to address the second bottleneck, curation. Computational methods are currently being developed that may help with curation, such as those being developed by InSilicoDB (13); however, these efforts are limited by inadequate user-supplied annotation and the lack of a controlled vocabulary to describe experimental samples.

New microarray and RNA-seq technologies are measuring different parts of the transcriptome, including miRNAs, long non-coding RNAs and exons. As the barcode methods are empirically driven, there currently are not enough data to develop barcodes for these technologies, but there will be in the near future. When possible, barcodes will be developed for these techniques to help researchers obtain a more complete view of the human and murine transcriptomes in health and disease.

## SUPPLEMENTARY DATA

Supplementary Data are available at NAR Online.

## FUNDING

National Institutes of Health [CA132480 to M.J.Z., CA009363 to M.N.M., GM083084 and GM103552 to R.A.I. and 1G20RR030939]; and Loyola Institutional funds. Funding for open access charge: Loyola Institutional Funds.

*Conflict of interest statement*. None declared.
